# Evolutionary and Expression Analyses Show Co-option of *khdrbs* Genes for Origin of Vertebrate Brain

**DOI:** 10.3389/fgene.2017.00225

**Published:** 2018-01-04

**Authors:** Su Wang, Qingyun Yang, Ziyue Wang, Shuoqi Feng, Hongyan Li, Dongrui Ji, Shicui Zhang

**Affiliations:** ^1^Laboratory for Evolution and Development, Institute of Evolution and Marine Biodiversity, Ocean University of China, Qingdao, China; ^2^Laboratory for Evolution and Development, Department of Marine Biology, Ocean University of China, Qingdao, China

**Keywords:** *khdrbs*, co-option, evolution, brain development, zebrafish

## Abstract

Genes generated by whole genome duplications (WGD) can be co-opted by changing their regulation process or altering their coding proteins, which has been shown contributable to the emergence of vertebrate morphological novelties such as vertebrate cartilage. Mouse *khdrbs* genes, differing from its invertebrate orthologs, were mainly expressed in brain, hinting that *khdrbs* gene family as a member of genetic toolkit may be linked to vertebrate brain development. However, the evolutionary relationship between *khdrbs* gene family and vertebrate brain development is unclear. First, we analyzed the evolutionary history of *khdrbs* gene family in metazoans, and then investigated their expression patterns during early development and in adulthood of zebrafish. We found that the duplication of *khdrbs* gene family by WGD took place in zebrafish, and all zebrafish *khdrbs* genes were predominantly expressed in the substructures of brain during early development. Given the expression of invertebrate *khdrbs* gene in germ line, the distinct expression domains of zebrafish *khdrbs* genes in brain suggested that the duplicated *khdrbs* genes are co-opted for promoting the evolutionary origin of vertebrate brain.

## Introduction

One of the key questions of evolution is to answer how the morphological complexity takes place among extant animals including vertebrates. The subphylum Vertebrata is characterized by the appearance of critical morphological innovations such as an elaborate segmented brain, neural crest cells, neurogenic placodes, and endoskeleton (Shimeld and Holland, 2000). Several decades of developmental genetic studies have led to the important discovery that most animals from different taxa share a bunch of regulatory and tissue-specific genes known as “genetic toolkit,” which controls animal body pattern (Carroll, 2000). Therefore, the molecular phylogeny of these toolkit genes will provide us an insight into the evolutionary origin of morphological novelties. Whole genome duplication (WGD) could not only enrich the genomic complexity, but also generate new functions of duplicated genes by co-option (Ohno, 1970; True and Carroll, 2002; Dehal and Boore, 2005; Conant and Wolfe, 2008; Cañestro et al., 2013). WGD occurred around the origin of vertebrate lineage, has been shown to be related to vertebrate morphological innovations (Manzanares et al., 2000; True and Carroll, 2002). For example, the origin of vertebrate cartilage was found to be attributable to the duplication of chordate fibrillar *collagen* genes (Wada et al., 2006; Zhang et al., 2006), and the emergence of vertebrate vascular vessels to the duplication of *kank* genes (Hensley et al., 2016).

The family of KHDRBS protein (KH Domain-containing, RNA Binding, and Signal transduction associated protein), is characterized by the GSG (GRP33/SAM68/GLD-1) domain, also named STAR domain, which includes a single KH domain flanked by the conserved N-terminal QUA1 and C-terminal QUA2 (Di Fruscio et al., 1999; Lukong and Richard, 2003). KHDRBS proteins bind RNA through their KH domains, and participate in signal transduction (Frisone et al., 2015; Ehrmann et al., 2016). In nematodes, GLD-1, the ortholog of KHDRBS, was found to be localized in the germ cell cytoplasm, and indispensable for oogenesis and meiotic prophase progression, while it shows little roles in the male germ line or soma, although it can stimulate sex determination of males in the hermaphrodite germ line (Jones and Schedl, 1995; Lee and Schedl, 2001, 2010). In contrast, NSR, the ortholog of KHDRBS of fruit fly, was found to be predominantly localized in the nuclei of primary spermatocytes, and necessary for male reproduction by regulating some male fertility genes (Ding et al., 2010). In vertebrates, there are three members of KHDRBS, KHDRBS1 (also called Sam68), KHDRBS2 (also called SLM1), and KHDRBS3 (also called SLM2) which share many features such as RNA binding and signal transduction. Interestingly, *khdrbs1* knockout mice showed impaired fertility in males as a result of mRNA translational regulation defect during spermiogenesis (Paronetto et al., 2009; Ehrmann and Elliott, 2010; Frisone et al., 2015), and KHDRBS3 was also involved in spermatogenesis via directly binding to the genes essential for male gametogenesis (Zhang et al., 2009). This function of KHDRBS1 and KHDRBS3 in mice is apparently analogous to their invertebrate orthologs GLD-1 and NSR in term of regulation of gametogenesis. On the contrary, the three members of vertebrate *khdrbs* family appear to be expressed more specifically in the brain. In mouse, *khdrbs1* and *khdrbs3* were predominantly expressed in the brain and testis, but *khdrbs2* was exclusively expressed in the brain (Ehrmann et al., 2016). Moreover, although *khdrbs1* KO male mice showed male infertility, behavioral deficits and poor motor control (Ehrmann et al., 2013), *khdrbs2* KO mice as well as *khdrbs1* and *khdrbs2* double KO mice both exhibited defects in cerebellar morphogenesis (Iijima et al., 2014), and *khdrbs3* KO mice displayed synaptic plasticity and behavioral defects (Traunmüller et al., 2016). These data together suggest that *khdrbs* gene family, as a member of genetic toolkit, may be linked to vertebrate brain development. However, the evolutionary relationship between *khdrbs* gene family and vertebrate brain development is still a mystery.

The aim of this study is thus to answer this question by taking advantage of the zebrafish (*Danio rerio*) model, which has a brain resembling that of humans in both basic structures and functional capacities (Tropepe and Sive, 2003). We first analyzed the molecular evolution of *khdrbs* gene family in representative metazoan taxa, and then examined the expression patterns of *khdrbs* during early development and in adulthood of zebrafish. We found that *khdrbs* gene family was expanded by WGD in zebrafish, and all zebrafish *khdrbs* genes were predominantly expressed in the substructures of brain during early development. Given that these substructures are vertebrate-specific trait, the distinct expression domains of *khdrbs* genes in zebrafish suggested that *khdrbs* gene family was co-opted for vertebrate brain development after WGD events around the split of Vertebrata.

## Materials and Methods

### Zebrafish Strain and Embryos

The AB strain zebrafish were cultured at 28 ± 1°C. Embryos were cultured in E3 medium consisting of 5 mM NaCl, 0.17 mM KCl, 0.33 mM CaCl_2_, and 0.33 mM MgSO_4_. For whole-mount *in situ* hybridization (WISH), 0.0045% 1-phenyl-2-thiourea was added into E3 medium to prevent embryos from pigmentation started 24 h post-fertilization (hpf). Different stages of embryos were sorted and fixed following the guide of Kimmel et al. (1995).

### Sequence Retrieval and Bioinformatics Analysis

KHDRBS protein sequences of most major metazoan taxa were obtained from NCBI and Ensembl database. Other KHDRBS protein sequences were identified by a BLASTp search with a query sequence (human KHDRBS1 protein). The sequences used were listed in Supplementary Table 1. Sequence alignment was performed by ClustalW method in MegAlign v7.1.0. To acquire the best evolutionary model for phylogenetic inference, we used default parameters in MEGA7 to conduct a best-fit protein model test. According to calculated BIC (Bayesian information criterion) scores for every model, we chose LG + G model which had the lowest score for further phylogenetic analysis. After that, rooted phylogenetic trees were constructed by Bayesian analysis and maximum likelihood (ML) method. These two methods were conducted with MrBayes v3.2.6 and PHyML website, respectively. ML phylogenetic analysis used LG + G model and set bootstrap as 1,000 replicates. Bayesian analysis was performed with following parameters: ngen = 2,000,000, nruns = 2, nchains = 4, aamodel = fixed (LG), rates = gamma, samplefreq = 1,000, burninfrac = 0.25. Finally, phylogenetic trees obtained were viewed and modified with FigTree v1.4.2. Synteny data of *khdrbs* genes were collected using tools available from Ensembl and NCBI database. Gene structural features of those chosen transcripts were analyzed using data from Ensembl database. Characteristics of the protein domain were predicted by SMART website.

### Gene Cloning, WISH, Cryosection, and qRT-PCR

Fragments of zebrafish *khdrbs* genes were amplified with specific primers (**Table 1**) that were designed using Primer Premier 5.0 based on existing sequences from NCBI. The purified PCR products were sub-cloned into vector pGEM-T, which was sequenced to verify inserts orientation.

**Table 1:** Primers used for specific antisense probe.

Gene	GenBank accession no.	Forward primer (5′–3′) Reverse primer (5′–3′)
*khdrbs1a*	NM_130925.1	CCTCAGGGCAGCACCATCAA
		CGCCACCTTCCCAGTCATCTT
*khdrbs1b*	NM_213235.1	TTAACTTTGTTGGGAAGATCCTTG
		GCTGCTTGTGGCTGACTGTAGTA
*khdrbs2*	NM_001077290.1	AAAAGTTTGAAGGCGACGAGTTG
		CTTGTGCGTGGCTTGCAGTG
*khdrbs3*	XM_009298280.2	TGTGCTTATTGAGGTGTTTGCTCC
		CGCTTTGTTGCGGTTGTTTGT

Digoxigenin (DIG)-labeled *khdrbs* antisense riboprobes were synthesized with linearized vectors (digested by *Nco*I restriction enzyme) and Sp6 RNA polymerase through *in vitro* transcription, while synthesis of sense riboprobes used *Sal*I restriction enzyme and T7 RNA polymerase. WISH experimental procedures followed the protocol described by Thisse and Thisse (2008). After staining, the embryos were fixed with 4% paraformaldehyde, rinsed with 70% ethanol and then mounted in glycerin for imaging. The embryos were also washed with PBS, soaked in 30% sucrose (diluted in PBS) overnight and cryosectioned.

Quantitative real-time PCR (qRT-PCR) was used to test the expression patterns of *khdrbs* in the different tissues of adult zebrafish. Total RNAs were extracted from the tissues gill, eye, brain, intestine, liver, heart, muscle, skin, spleen, testis, and ovary with TRIzol^TM^ (Invitrogen) and purified using Total RNA Kit I (OMEGA Bio-Tek). For each tissue, 1 μg RNA was used for next reverse transcription. The cDNAs were reverse transcribed using M-MLV reverse transcriptase (TaKaRa) and Oligo (dT) primers as guided by the manufacturer’s instructions. *β-actin* and *EF1-α* were chosen as control to standardize the results by eliminating variations in mRNA and cDNA quantity and quality. qRT-PCR was conducted using the gene-specific primers (**Table 2**) on the ABI 7500 real-time PCR system (Applied Biosystem) with the 2× SYBR Premix Ex Taq^TM^ Kit (TaKaRa). A total of 0.5 μl cDNA was used as template in each replicate. Reaction conditions were: 95°C for 15 s as stage 1, then 40 cycles of 95°C for 15 s, 60°C for 15 s, and 72°C for 35 s as stage 2. The expression level of *khdrbs* genes relative to that of the housekeeping genes *β-actin* and *EF1-α* was calculated by the comparative threshold cycle (CT) method (2^-ΔΔCt^). The experiments were performed in triplicate, and each replicate was from three experiments, i.e., the indicated replicates were both technical and biological replicates.

**Table 2:** Primers used for qRT-PCR.

Gene	GenBank accession no.	Forward primer (5′–3′) Reverse primer (5′–3′)
*khdrbs1a*	NM_130925.1	CCTCAGGGCAGCACCATCAA
		TCCATACCTTCCACAGGCATCA
*khdrbs1b*	NM_213235.1	GAAAGGATTCTCATACCAGTCAAACA
		CAGGAGCAAACACCTCAATAAACAC
*khdrbs2*	NM_001077290.1	CAAGGGTTCAATGAGGGATAAAGG
		TGTGGAGGTCAGCCGCAATC
*khdrbs3*	XM_009298280.2	TCCATGTGCTTATTGAGGTGTTTGC
		TGTTCCCGGCGCTGATGTT
*β-actin*	NM_131031.1	GGTATTGTGATGGACTCTGGTGAT
		TCGGCTGTGGTGGTGAAG
*EF1-α*	NM_131263.1	GACTGGTGTCCTCAAGCCTGGTATG
		CAATCCAGCACTGGGGCGTAAC

## Results

### Three Groups of *khdrbs* Genes in Vertebrates

**FIGURE 1:**
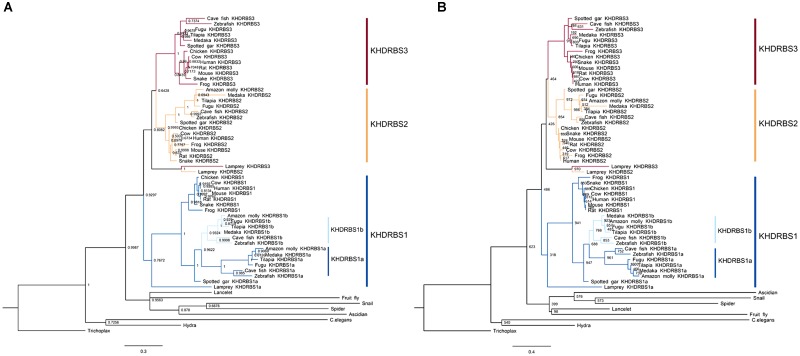
Phylogenetic analyses of KHDRBS proteins constructed by **(A)** Bayesian method and **(B)** maximum likelihood (ML) method. Numbers at each node suggest Bayesian posterior probability (pp) values based on two million runs and ML bootstrap values based on 1,000 replicates, respectively. Both trees are rooted with *Trichoplax*.

Recent studies have revealed that changes of key genetic toolkit at cellular and developmental level can generate novel characters in morphological structures. To better understand, it we need to dig into the evolutionary history of these critical genes. Considering that DNA sequences are more vulnerable and variable to selection pressure during long evolutionary history, we used KHDRBS protein sequences of major metazoan taxa for later analyses. We acquired multiple KHDRBS protein sequences mainly from NCBI and Ensembl. These sequences were aligned by ClustalW algorithm for next phylogeny analyses. We constructed phylogenetic trees by MrBayes (**Figure 1A**) and ML methods (**Figure 1B**). These two phylogenetic trees were generally consistent with each other. The analyses revealed that KHDRBS was traced back to *Trichoplax adhaerens* and *Hydra vulgaris*, the basal metazoans.

**FIGURE 2:**
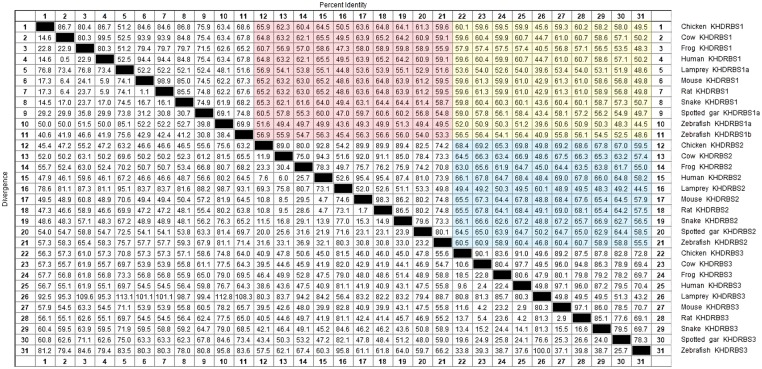
KHDRBS protein sequences alignment of major vertebrates. Red area represents the percent identity between KHDRBS1 and KHDRBS2. Yellow area means the percent identity between KHDRBS1 and KHDRBS3. And blue area shows the percent identity between KHDRBS2 and KHDRBS3. Protein sequences are aligned by ClustalW method in MegAlign.

The invertebrate KHDRBS proteins formed a single clade, positioned at the base of vertebrate KHDRBS proteins. The overall phylogenetic relationship of KHDRBS well reflects the classic phylogeny of metazoan taxa. In contrast to only one KHDRBS group in invertebrates, there were three distinct clades of KHDRBS proteins in vertebrates, KHDRBS1, KHDRBS2, and KHDRBS3 (**Figure 1**), consistent with recent reports that humans, mice, and birds have three KHDRBS genes (Artzt and Wu, 2010). Apparently, the emergence of these three clades occurred around the split of subphylum Vertebrata, implicating that the three KHDRBS clades originated from sequential WGD events. Notably, absence of KHDRBS3 was also observed in Amazon molly (*Poecilia formosa*), suggesting that gene loss event may have happened during evolution process. In addition, KHDRBS protein sequences alignment of major vertebrates (**Figure 2**) showed that KHDRBS2 and KHDRBS3 shared higher identity (range from 44.5 to 69.8%) than that between KHDRBS1 and KHDRBS2 (range from 43.6 to 65.9%) or KHDRBS1 and KHDRBS3 (range from 39.6 to 61.3%), which meant KHDRBS2 and KHDRBS3 formed a more close evolutionary relationship.

### Duplication of *khdrbs1* Genes in Teleosts

**FIGURE 3:**
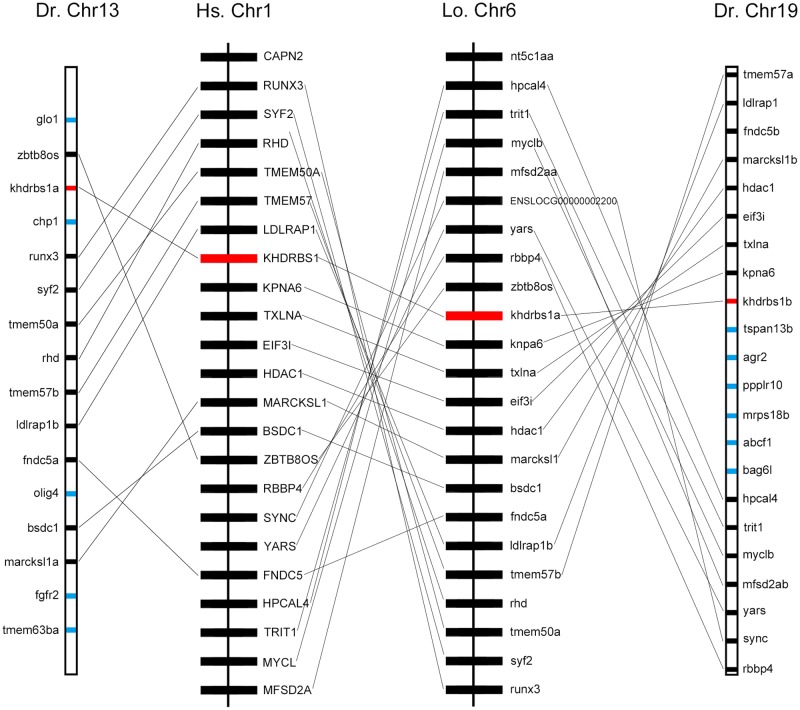
Syntenic map of the genomic segment where *khdrbs1* resides in the chromosomes of zebrafish, spotted gar, and humans. *khdrbs1* genes are highlighted in red and the synteny are linked with straight line. Other genes are noted with blue. The illustration of genes and their sizes are not proportional to the actual length of chromosome. Dr, zebrafish; Hs, human; Lo, spotted gar; Chr, chromosome.

We found that *khdrbs1* genes in teleost were clustered into two sub clades, as *khdrbs1a* and *khdrbs1b*. Compared to tetrapod *khdrbs1* genes, *khdrbs1a* and *khdrbs1b* formed a more close branch with a sister clade spotted gar (**Figure 1**). Spotted gar has an important evolutionary status because it diverged from teleost before gene duplication happened in teleost (Braasch et al., 2016). Therefore, this indicated that *khdrbs1a* and *khdrbs1b* emerged simultaneously within teleost lineage, i.e., they were generated by teleost-specific genome duplication (Hoegg et al., 2004; Jaillon et al., 2004). As phylogenetics, albeit widely analyzed, may be still short of enough reliability to elucidate gene orthologous relationships due to sophisticated situations like gene loss or gene expansion in specific lineage, we thus performed a syntenic analysis among zebrafish *khdrbs1*, spotted gar *khdrbs1a* and human *khdrbs1* genes, which represented teleost and tetrapod, respectively (**Figure 3**), to further confirm the relationship between *khdrbs1a* and *khdrbs1b*. The results revealed that the flanking genes beside both *khdrbs1a* and *khdrbs1b* could be found in the neighboring region around spotted gar and human *khdrbs1*, which meant that *khdrbs1* in zebrafish was orthology to spotted gar and human *khdrbs1* and remained an evolutionarily conserved synteny.

**FIGURE 4:**
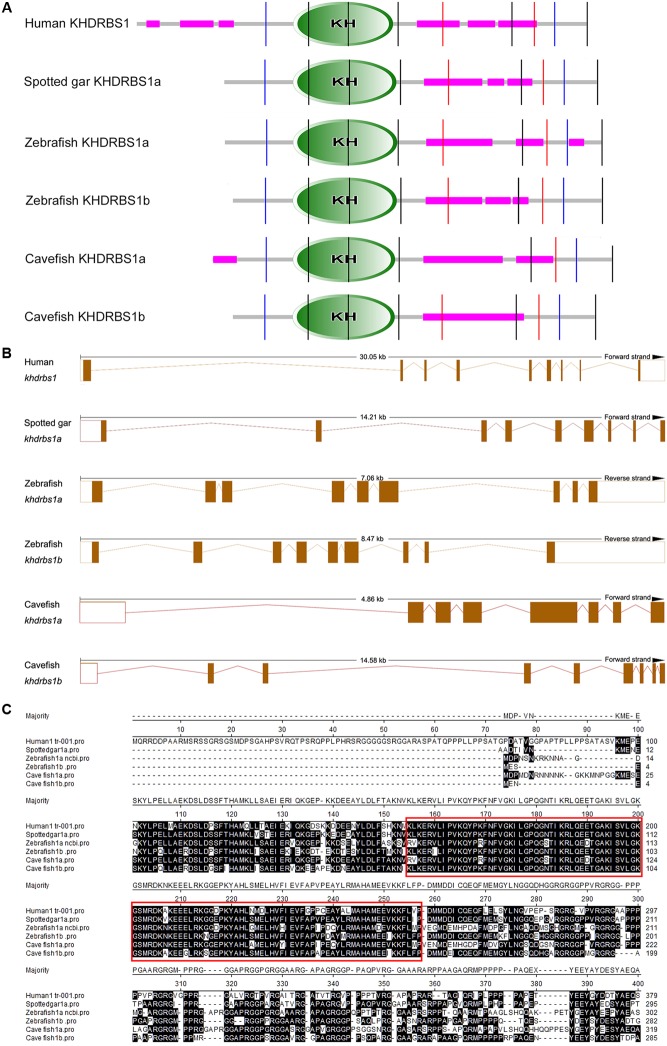
Bioinformatic analysis of KHDRBS1 proteins in cavefish, human, spotted gar, and zebrafish. **(A)** Protein domain structure predictions of KHDRBS1 by SMART. Purple bars represent low complexity regions. Vertical colored lines indicate the intron positions. KH domains are shown in oval shapes with “KH”. **(B)** Intron–exon structures of *khdrbs1*. The solid boxes denote the gene coding regions, and the empty boxes mean untranscribed regions. Dotted lines represent the introns. **(C)** Protein sequences alignment of KHDRBS1. Protein sequences are aligned by ClustalW method in MegAlign. The highly conserved part is noted by red rectangles.

To pinpoint the relationships between the duplications of teleost *khdrbs1* genes, we conducted protein domain analysis by SMART. We found that when compared with human KHDRBS1, both zebrafish KHDRBS1a and KHDRBS1b possessed similar protein domains, though they shared more similarities to spotted gar KHDRBS1a (**Figure 4A**). Additionally, the differences between genomic intron–exon structures of zebrafish *khdrbs1a* and *khdrbs1b* were also analyzed. Both *khdrbs1a* and *khdrbs1b* had an intron–exon structure similar to that of other species (**Figure 4B**), with the highly conserved region within the KH domains (**Figure 4C**).

### Similar but Distinct Expression Patterns of Zebrafish *khdrbs* Genes

WGD commonly leads to the functional specialization of paralogous genes, or gene co-option, and contributes to vertebrate morphological innovations. Recently, *khdrbs1a* and *khdrbs1b* were found both mainly expressed in the brain primordium of zebrafish embryos, hinting at the clue that *khdrbs* genes may play critical roles in vertebrate brain development. We thus hypothesized that investigating the gene expression patterns of the four zebrafish *khdrbs* genes might illuminate the origin of the vertebrate brain during evolution.

**FIGURE 5:**
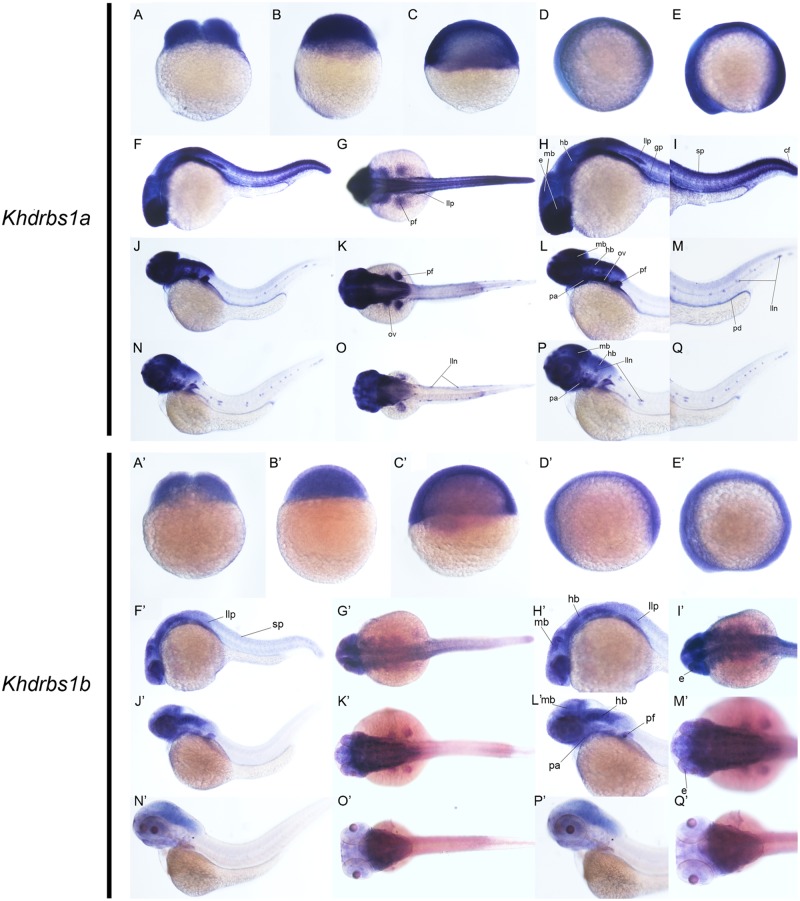
Gene expression patterns of zebrafish *khdrbs1a* and *khdrbs1b* genes during early development detected by WISH. Stages of embryonic development: 0.45 hpf **(A,A′)**, 3 hpf **(B,B′)**, 6 hpf **(C,C′)**, 10 hpf **(D,D′)**, 14 hpf **(E,E′)**, 24 hpf **(F–I, F′–I′)**, 48 hpf **(J–M, J′–M′)**, and 72 hpf **(N–Q, N′–Q′)**. Expression sites are labeled with abbreviation as follows. cf, caudal fin; e, eye; gp, gonad primordium; hb, hindbrain; lln, lateral line neuromast; llp, lateral line primordium; mb, midbrain; ov, otic vesicle; pa, pharyngeal arch; pd, pronephric ducts; pt, pectoral fin; sp, spinal cord.

**FIGURE 6:**
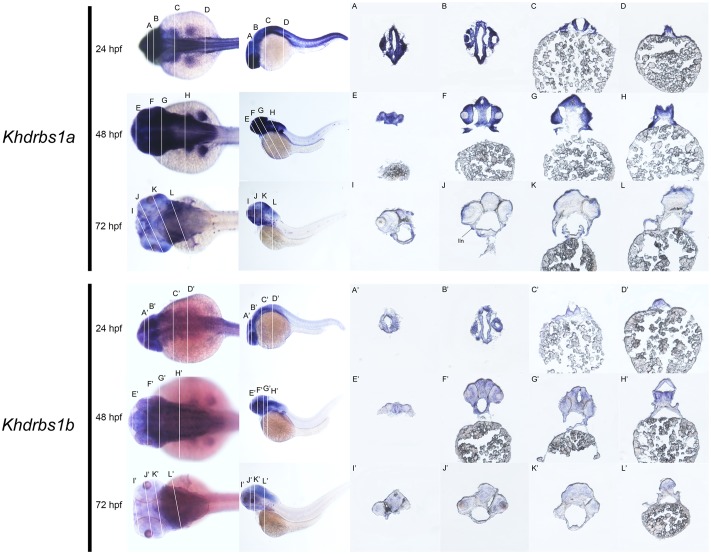
Gene expression domains of zebrafish *khdrbs1a* and *khdrbs1b* genes in brain detected by WISH and cryosection. Transverse sections of brain structures: telencephalon **(E,I,E′,I′)**, diencephalon **(F,J,F′,J′)**, midbrain **(G,K,G′,K′)** and hindbrain **(C,D,H,L,C′,D′,H′,L′)**. Brain structure of zebrafish at 24 hpf **(A,B,A′,B′)**. Straight lines, representing slice positions, are marked by English letters corresponding to the section images. Expression sites are labeled with abbreviation as follows. lln, lateral line neuromast.

**FIGURE 7:**
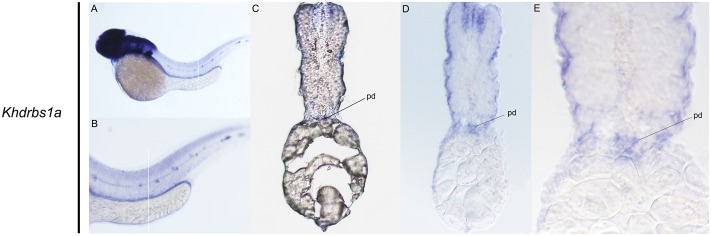
Gene expression domains of zebrafish *khdrbs1a* gene in pronephric ducts detected by WISH and cryosection at 48 hpf. Straight lines represent slice positions **(A,B)**. Transverse sections of zebrafish pronephric ducts at 48 hpf **(C–E)**. Expression sites are labeled with abbreviation as follows. pd, pronephric ducts.

Zebrafish have two *khdrbs1* genes, *khdrbs1a* and *khdrbs1b*. Different from control group (Supplementary Figures 1A–F), both *khdrbs1a* and *khdrbs1b* were pan-expressed in the blastoderm and the embryo *per se* (**Figures 5A–E,A′–E′**). At 24 hpf, *khdrbs1a* expression was detected in the brain (**Figures 6A–D**), eye, spinal cord, lateral line primordium, gonad primordium, pectoral fin, and caudal fin (**Figures 5F–I**), while *khdrbs1b* expression was relatively weak in the brain (**Figures 6A′–D**′), eye, lateral line primordium, and spinal cord (**Figures 5F′–I′**). By 48 hpf (**Figures 5J–M**) and 72 hpf (**Figures 5N–Q**), *khdrbs1a* expression in the brain (**Figures 6E–L**) and pectoral fin enhanced, and the signal at the lateral line neuromast was evident. Additional expressions were also observed in the pharyngeal arch, pronephric ducts (**Figure 7**) and otic vesicle, but the expression signals in the spinal cord and caudal fin apparently faded. Compared to *khdrbs1a*, *khdrbs1b* was not expressed in the lateral line by 48 hpf (**Figures 5J′–M′**); instead, its expression was restricted to the brain (**Figures 6E′–H′**), eye, pharyngeal arch and pectoral fin, and this expression pattern remained till 72 hpf (**Figures 5N′–Q′**, **6I′–L′**).

**FIGURE 8:**
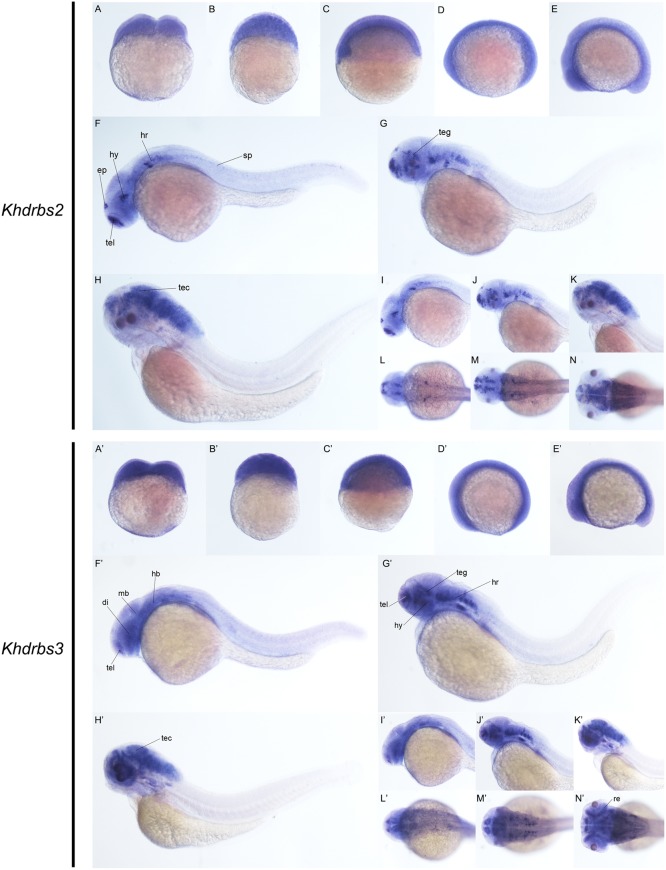
Gene expression patterns of zebrafish *khdrbs2* and *khdrbs3* genes during early development detected by WISH. Stages of embryonic development: 0.45 hpf **(A,A′)**, 3 hpf **(B,B′)**, 6 hpf **(C,C′)**, 10 hpf **(D,D′)**, 14 hpf **(E,E′)**, 24 hpf **(F,I,L,F′,I′,L′)**, 48 hpf **(G,J,M,G′,J′,M′)**, and 72 hpf **(H,K,N,H′,K′,N′)**. Expression sites are labeled with abbreviation as follows. di, diencephalon; ep, epiphysis; hb, hindbrain; hr, hind rhombomeres; hy, hypothalamus; mb, midbrain; re, retina; sp, spinal cord; tec, tectum; teg, tegmentum; tel, telencephalon.

**FIGURE 9:**
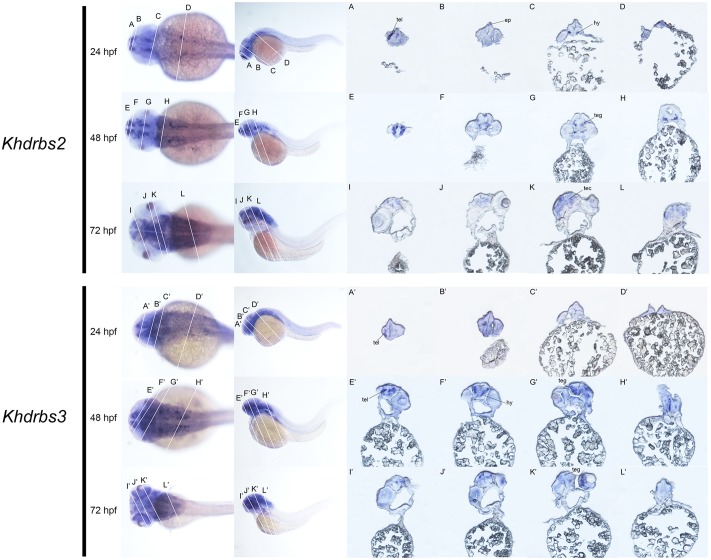
Gene expression domains of zebrafish *khdrbs2* and *khdrbs3* genes in brain detected by WISH and cryosection. Transverse sections of brain structures: telencephalon **(A,E,I,A′,E′,I′)**, diencephalon **(B,F,J,B′,F′,J′)**, midbrain **(C,G,K,C′,G′,K′)**, and hindbrain **(D,H,L,D′,H′,L′)**. Straight lines, representing slice positions, are marked by English letters corresponding to the section images. Expression sites are labeled with abbreviation as follows. ep, epiphysis; hy, hypothalamus; tec, tectum; teg, tegmentum; tel, telencephalon.

We then examined the expression patterns of zebrafish *khdrbs2* and *khdrbs3* expression. At the early developmental stages, both *khdrbs2* and *khdrbs3*, like *khdrbs1*, were pan-expressed in the blastoderm and the embryo *per se* (**Figures 8A–E,A′–E′** and Supplementary Figures 1G–L). However, *khdrbs2* and *khdrbs3* genes both later generated more specific expression patches in the brain, forming a sharp contrast to that of *khdrbs1*. By 24 hpf (**Figures 8F,I,L, 9A–D**), *khdrbs2* was expressed in the telencephalon, epiphysis, hypothalamus, hind rhombomeres, and spinal cord. Signals in these regions gradually expanded in the midbrain and hindbrain with larva development. At 48 hpf (**Figures 8G,J,M, 9E–H**) and 72 hpf (**Figures 8H,K,N, 9I–L**), expression of *khdrbs2* was primarily restricted to the tegmentum and tectum, respectively, and expression in the spinal cord disappeared. More interestingly, *khdrbs3* signal was observed in the telencephalon, diencephalon, midbrain, and hindbrain at 24 hpf (**Figures 8F′,I′,L′, 9A′–D′**), and then became localized intensely in the telencephalon, hypothalamus, tegmentum, and hind rhombomeres (**Figures 8G′,J′,M′, 9E′–H′**). By 72 hpf (**Figures 8H′,K′,N′, 9I′–L′**), *khdrbs3* expression spread out around the midbrain and hindbrain, with additional signals emerging in the retina and tectum.

**FIGURE 10:**
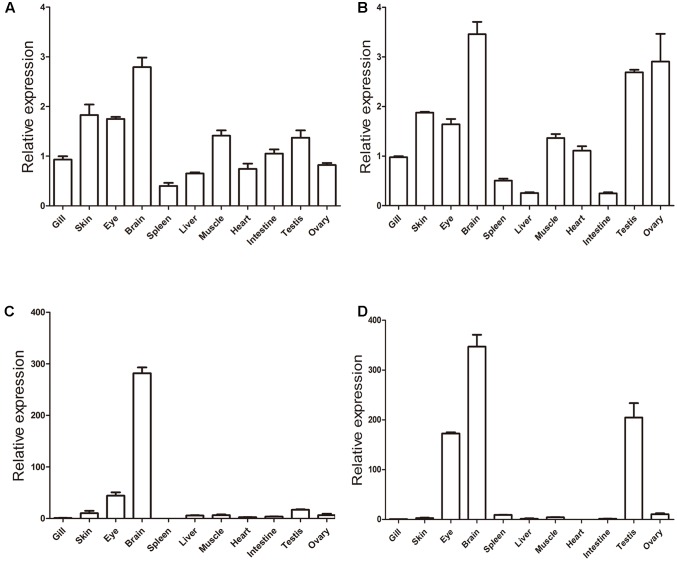
Gene expression patterns of zebrafish **(A)** *khdrbs1a*, **(B)** *khdrbs1b*, **(C)** *khdrbs2*, and **(D)** *khdrbs3* in different tissues. *β-actin* is used as internal control for normalization. Relative expression data is calculated by the method of 2^-ΔΔCt^. Vertical bars represent the mean ± standard deviation (SD) (*n* = 3). Data are from three independent experiments which were performed in triplicate.

Finally, we carried out qRT-PCR to explore the expression profiles of *khdrbs* in adult zebrafish (**Figure 10** and Supplementary Figure 2). As shown in **Figures 10A,B**, both *khdrbs1a* and *khdrbs1b* shared similarities in their expression profiles. They were both detected in all the tissues tested, with the highest expression levels in the brain. The obvious difference between *khdrbs1a* and *khdrbs1b* was that the latter gene had higher expression in the gonads. In contrast, the expression of *khdrbs2* and *khdrbs3* became more distinct: both *khdrbs2* and *khdrbs3* signal was predominantly detected in the brain, with lower expression in the eye and testis (**Figures 10C,D**). These indicated that these genes are mainly expressed in the brains of adult zebrafish in a tissue-specific fashion, consistent with their expression patterns in embryogenesis.

## Discussion

One goal for evolutionary biology is to decipher the mystery underlying morphological novelties and complexity of extant organisms, especially vertebrates. As the largest and most diverse lineage in chordate, Vertebrata is characterized by several key morphological characteristics such as a tripartite brain. Such morphological characteristics are usually originated from variation of the related genetic toolkits. Hence, expression pattern and evolutionary analyses of key genes in these toolkits can deepen our understanding of the emergence of morphological innovations during evolution. In this study, we first analyzed the molecular evolution of *khdrbs* gene family, and then investigated zebrafish *khdrbs* genes expression patterns during embryonic development and in adulthood of zebrafish. The results indicated that after vertebrate WGD, *khdrbs* gene family was co-opted for brain development, and the duplication and diversification of *khdrbs* genes may promote the origin of vertebrate elaborate brains during evolution.

### Evolutionary History of *khdrbs* Genes

The *khdrbs* genes are evolutionarily conserved. We found that *khdrbs* date far back to the basal metazoans such as placozoa and cnidarian. Except for fruit fly, all the invertebrates analyzed possess a single *khdrbs* gene in each species. In fruit fly, there are five *khdrbs* genes, including *qkr54B*, *qkr58E-1*, *qkr58E-2*, *qkr58E-3*, and *nsr* (Ding et al., 2010; Volk, 2010), which are all localized on a region of the chromosome 2R, and clustered into one branch (Supplementary Figure 3), suggesting that they were generated by local tandem gene duplication of the ancestral *khdrbs* gene. Based on their expression patterns and tandem duplicated gene traits (Fan et al., 2008; Zhou et al., 2008; Volk, 2010), we speculated that these five genes perform similar biological functions. In contrast, three *khdrbs* genes, *khdrbs1*, *khdrbs2*, and *khdrbs3*, are identified in all the vertebrates examined, including lamprey (Xu et al., 2016). The birth of these three *khdrbs* gene clades coincides with the origin of vertebrates. As there existed two rounds (2R) of WGD happened in the vertebrate ancestors, it is thus expected that four *khdrbs* genes would be generated by these WGD. Interestingly, there are only three *khdrbs* genes identified so far in vertebrates. It is thus highly likely that only one copy out of the two *khdrbs* genes generated by the first round WGD underwent further duplication, thereby leading to the appearance of three *khdrbs* genes in vertebrates. This seems supported by the facts that KHDRBS2 and KHDRBS3 were grouped together, forming a sub-clade in the phylogenetic trees (**Figure 1**), and shared higher identity (**Figure 2**). However, the possibility cannot be ruled out that this may be due to gene loss as observed in Amazon molly. Additionally, we found duplication of *khdrbs1* in teleost lineage. This duplication happened between the split of spotted gar and bony fish, agreeing with the third rounds (3R) of WGD in bony fish (Braasch et al., 2016).

**FIGURE 11:**
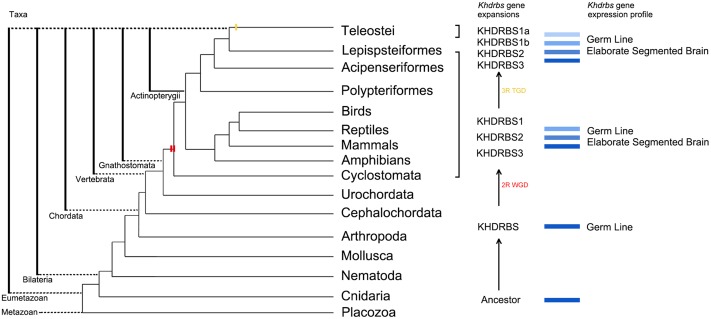
Molecular evolution of *khdrbs* genes and the origin of vertebrate tripartite brain. The evolutionary relationships of metazoan and Vertebrata are based on our analyses above and recent phylogenomic analyses. Two red bars represent the 2R WGD events happening around the origin of vertebrate linage. The yellow one indicates teleost specific WGD event. Those blue rectangles represent the process of *khdrbs* gene duplication and diversification. The branch lengths are not proportional to the time of diversification.

On the basis of our analyses on *khdrbs* gene family evolution and the mainstream opinions in WGD, we propose an evolutionary model for *khdrbs* gene family, as depicted in **Figure 11**. In brief, an invertebrate *khdrbs* gene underwent duplication once, and one copy of the resulting genes, as exemplified by *khdrbs1*, remained unchanged, while the other copy underwent duplication once more, thereby creating the vertebrate *khdrbs1*, *khdrbs2*, and *khdrbs3*. In teleost, the *khdrbs1* was further duplicated through bony fish specific WGD, forming *khdrbs1a* and *khdrbs1b*. In some species like Amazon molly, *khdrbs3* is lost in a lineage-specific manner.

### Distinct and Overlapping Expression Profiles of Zebrafish *khdrbs* Genes

Expression pattern of genes is often associated with their functions in early development. We found all the zebrafish *khdrbs* genes were primarily expressed in the brain, while *khdrbs1a* was expressed in extra domains like the lateral line primordium, gonad primordium, and pronephric ducts. In adult fish, all the *khdrbs* also showed strong expression in the brain. Additionally, *khdrbs1a*, *khdrbs1b*, and *khdrbs3* were found expressed abundantly in the gonads. These overlapping but distinct expression patterns may be a reflection of the sub-functionalization and/or neo-functionalization of gene duplicates after WGD. The split of bony fish from tetrapod happened around 450 million years ago. The fact that all the four *khdrbs* genes are retained in bony fish since the split may be because the KHDRBS proteins are signal-transduction related molecules (Putnam et al., 2008). Retained *khdrbs* genes may enrich their functions through changes in their expression patterns, which may then be co-opted in regulation network to generate physiological and morphological evolution.

### Co-option of Vertebrate *khdrbs* Genes for Brain Development

Species diverges from common ancestors through changes in their DNA. Duplicated genes that impart new roles from their ancestors can be co-opted by changing their regulation process or altering their coding proteins to promote developmental and physiological features (Carroll, 2000; True and Carroll, 2002; Conant and Wolfe, 2008). A complex tripartite brain especially telencephalon is a key trait of vertebrates (Murakami et al., 2005; Sugahara et al., 2013, 2016). The highly plastic vertebrate brain influences all the basic biological functions, advance order processing and behavior control (Koscik and Tranel, 2012). As the most important part in organisms, however, it is hard to trace brain evolution because brains themselves are rarely fossilized. Hence, to explore the evolutionary origin of vertebrate brain needs to rely on molecular evolution and developmental studies.

Currently, mouse *khdrbs* genes were found expressed primarily in the brain, differing from that of the invertebrate orthologs, and *khdrbs* KO mice exhibited brain defects, indicating that *khdrbs* genes may be in the genetic toolkit correlated to vertebrate brain development (Ehrmann et al., 2013). In invertebrates, *khdrbs* genes were found expressed specifically in germ line (Lee and Schedl, 2001; Ding et al., 2010), so as zebrafish *khdrbs1a*, which was also expressed in the gonad primordium. Thus we surmise that one of the functions about *khdrbs* genes is to regulate gonad genesis. In addition to expression in gonads, zebrafish *khdrbs* genes were also found expressed in new domains such as the substructures of brain, especially in telencephalon. Telencephalon is an utterly vertebrate innovation (Murakami et al., 2005; Šestak and Domazet-Lošo, 2015). Considering that KHDRBS proteins act as splicing regulator molecules to control alternative splicing, and the brains have the largest amount of alternative splicing in the body, this distinct expression pattern of *khdrbs* genes gives a hint that the duplicated *khdrbs* genes were co-opted for motivating the evolutionary origin of vertebrate brain. Further studies on the expression and functions of *khdrbs* genes in other representative species within vertebrate lineage will better clarify the roles of KHDRBS played in brain evolution.

## Ethics Statement

All the zebrafish used in the experiments were treated in accordance with the guidelines of the Laboratory Animal Administration Law of China, with the permit number SD2007695 approved by the Ethics Committee of the Laboratory Animal Administration of Shandong Province.

## Author Contributions

SZ and DJ conceived and designed the experiment. SW, SF, and QY conducted the experiment. SW, HL, and DJ analyzed the data. SW, QY, and ZW prepared the figures and the tables. All the authors were involved in discussion and editing of the manuscript.

## Conflict of Interest Statement

The authors declare that the research was conducted in the absence of any commercial or financial relationships that could be construed as a potential conflict of interest.
