# Supplementary material for: Evolutionary and Expression Analyses Show Co-option of khdrbs Genes for Origin of Vertebrate Brain
Source: Front Genet. 2018 Jan 4;8:225. doi: 10.3389/fgene.2017.00225 (PMC5758493; doi:10.3389/fgene.2017.00225)
Supplement: Supplementary file 1 [file Data_Sheet_1.doc]

***Supplementary Material***

**Evolutionary and Expression Analyses Show Co-option of *khdrbs* Genes for Origin of Vertebrate Brain**

**Su Wang**

***Correspondence:**

Dongrui Ji

dongruij@yahoo.com

Shicui Zhang

sczhang@ouc.edu.cn


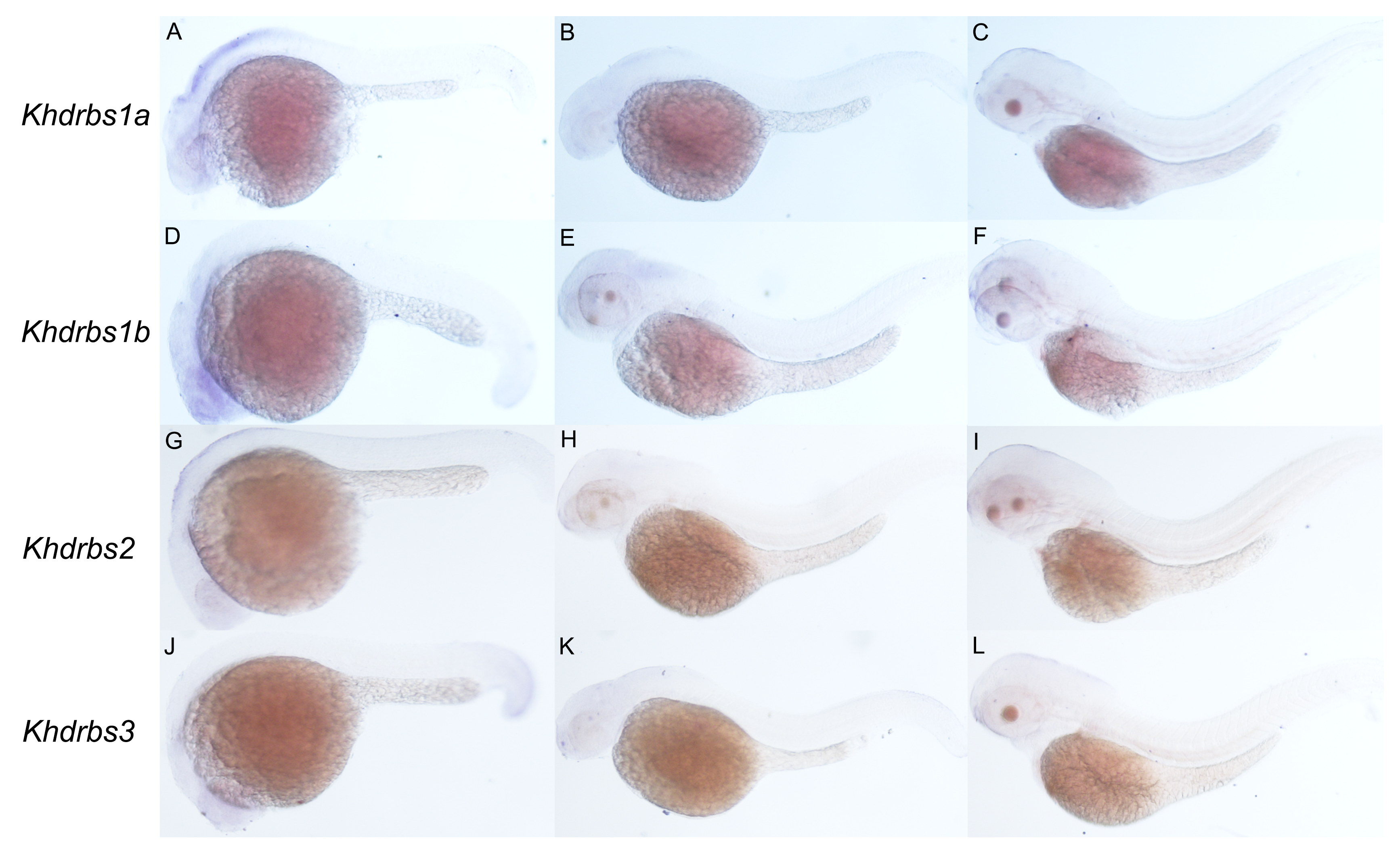


Supplementary Figure 1. Embryos hybridized with *khdrbs1a*, *khdrbs1b*, *khdrbs2* and *khdrbs3* sense riboprobes as control by WISH. Stages of embryonic development: 24 hpf **(A, D, G, J)**, 48 hpf **(B, E, H, K)** and 72 hpf **(C, F, I, L)**.


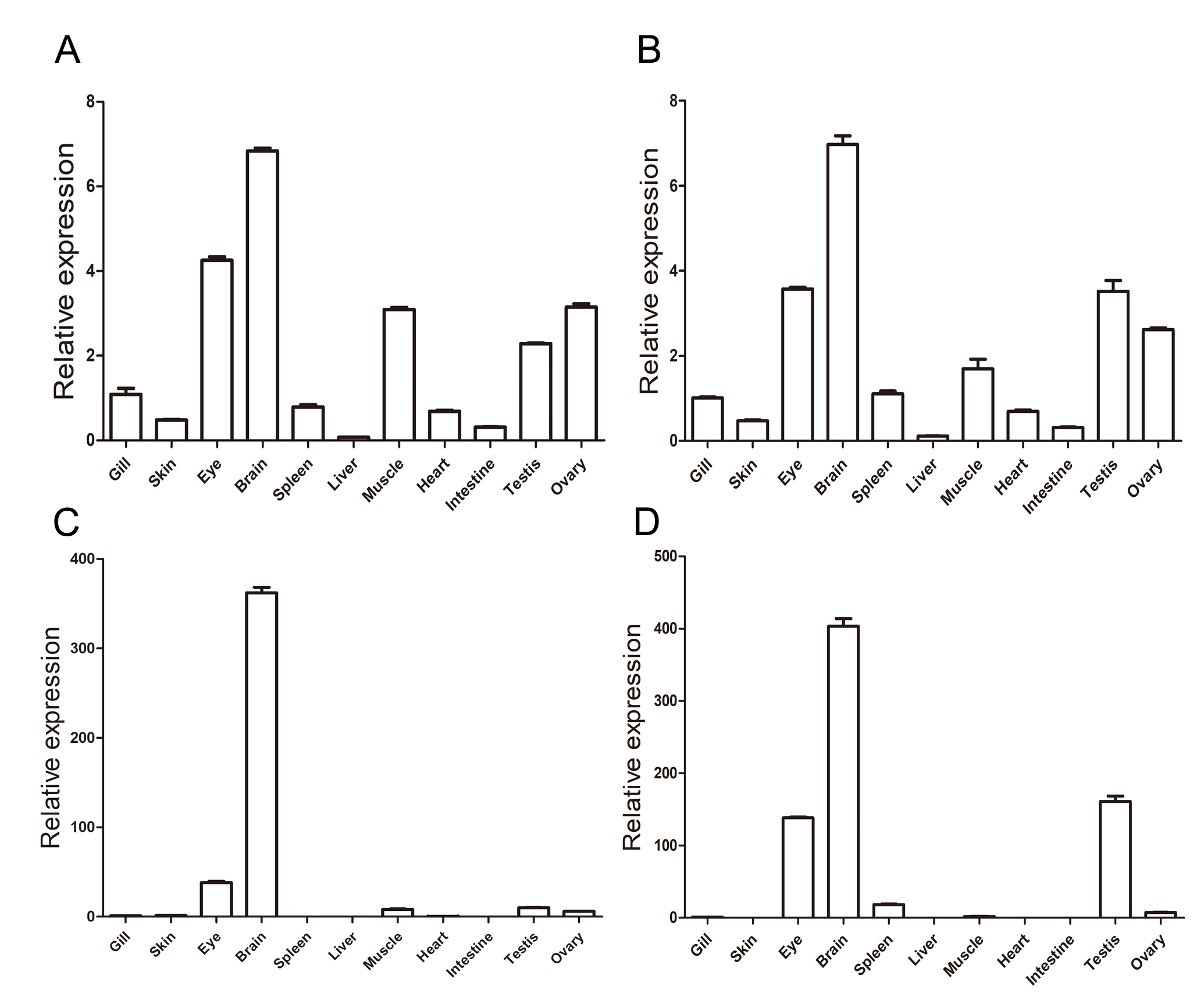


Supplementary Figure 2. Gene expression patterns of zebrafish **(A)** *khdrbs1a*, **(B)** *khdrbs1b*, **(C)** *khdrbs2* and **(D)** *khdrbs3* in different tissues. *EF1-α* is used as internal control for normalization. Relative expression data is calculated by the method of 2^-∆∆Ct^. Vertical bars represent the mean ± standard deviation (SD) (n = 3). Data are from 3 independent experiments which were performed in triplicate.


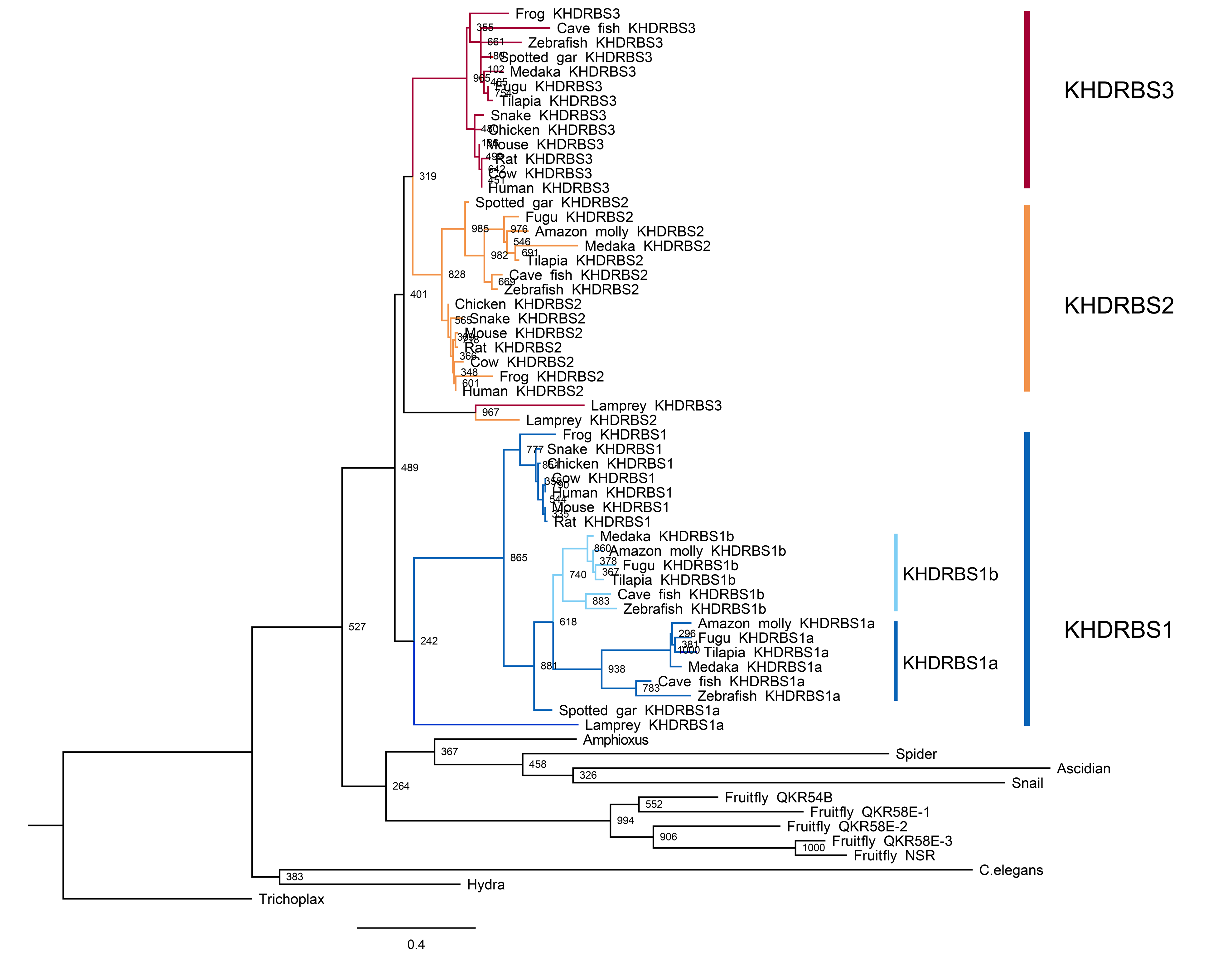


Supplementary Figure 3. Phylogenetic analyses of KHDRBS proteins constructed by Maximum Likelihood (ML) method. Numbers at each node suggest ML bootstrap values based on 1,000 replicates. The tree is rooted with *Tricoplax*.

Supplementary Table 1. KHDRBS protein sequences used for the phylogenetic analysis.

| Common name | Scientific name | KHDRBS1 or KHDRBS1a | KHDRBS1b | KHDRBS2 | KHDRBS3 |
| --- | --- | --- | --- | --- | --- |
| Amazon molly  Amphioxus | *Poecilia Formosa*  *Branchiostoma floridae* | XP_007549227.1  XP_002609602.1 | ENSPFOP00000012236 | ENSPFOP00000022461 |  |
| Ascidian | *Ciona intestinalis* | XP_018670868.1 |  |  |  |
| C.elegans | *Caenorhabditis elegans* | NP_492143.1 |  |  |  |
| Cow | *Bos taurus* | NP_001039907.1 |  | XP_010816377.1 | NP_001076981.1 |
| Cave fish | *Astyanax mexicanus* | ENSAMXP00000002899.1 | ENSAMXP00000012183.1 | ENSAMXP00000009846.1 | XP_007239295.1 |
| Chicken | *Gallus gallus* | NP_989561.1 |  | XP_426201.2 | XP_001231272.1 |
| Frog | *Xenopus tropicalis* | XP_012812472.1 |  | NP_001072215.1 | XP_002932263.1 |
| Fruitfly | *Drosophila melanogaster* | NP_611682.1 |  |  |  |
| Fugu | *Takifugu rubripes* | XP_011614545.1 | ENSTRUP00000025163.1 | ENSTRUP00000040274.1 | XP_003964226.1 |
| Human | *Homo sapiens* | NP_006550.1 |  | NP_689901.2 | AAH68536.1 |
| Hydra | *Hydra vulgaris* | XP_012559747.1 |  |  |  |
| Lamprey | *Petromyzon marinus* | ENSPMAP00000005023.1 |  | ENSPMAT00000004038.1 | ENSPMAT00000003043.1 |
| Medaka | *Oryzias latipes* | XP_004082415.1. | XP_020557443.1 | XP_020565313.1 | XP_004074298.1 |
| Mouse | *Mus musculus* | NP_035447.3 |  | NP_573498.1 | NP_034288.2 |
| Rat | *Rattus norvegicus* | NP_569089.1 |  | NP_579852.1 | NP_071585.1 |
| Snail | *Biomphalaria glabrata* | XP_013076155.1 |  |  |  |
| Snake | *Protobothrops mucrosquamatus* | XP_015669185.1 |  | XP_015671390.1 | XP_015682414.1 |
| Spider | *Parasteatoda tepidariorum* | XP_015911258.1 |  |  |  |
| Spotted gar | *Lepisosteus oculatus* | XP_006631296.1 |  | XP_015218627.1 | XP_006635718.1 |
| Tilapia | *Oreochromis niloticus* | XP_005453400.1 | XP_013125947.2 | ENSONIP00000012252.1 | XP_003443805.2 |
| Trichoplax | *Trichoplax adhaerens* | XP_002113951.1. |  |  |  |
| Zebrafish | *Danio rerio* | NP_571000.1 | NP_998400.1 | NP_001070758.1 | XP_009296555.1 |
